# Low APOA-1 Expression in Hepatocellular Carcinoma Patients Is Associated With DNA Methylation and Poor Overall Survival

**DOI:** 10.3389/fgene.2021.760744

**Published:** 2021-11-01

**Authors:** Yingyun Guo, Binglu Huang, Ruixue Li, Jiao Li, Shan Tian, Cheng Peng, Weiguo Dong

**Affiliations:** ^1^ Department of Gastroenterology, Renmin Hospital of Wuhan University, Wuhan, China; ^2^ Department of Gastroenterology, Macheng Renmin Hospital, Macheng, Huanggang, China; ^3^ Department of Infectious, Union Hospital, Tongji Medical College, Huazhong University of Science and Technology, Wuhan, China

**Keywords:** apolipoprotein A1 (APOA-1), hepatocellular carcinoma (HCC), DNA-methylation, prognosis, bioinformatic analysis

## Abstract

**Background:** Hepatocellular carcinoma (HCC) is the most frequent fatal malignancy, and it has a poor prognosis. Apolipoprotein 1 (APOA-1), the main protein component of high-density lipoproteins, is involved in numerous biological processes. Thus, this study was performed to detect the clinical significance of APOA-1 mRNA, APOA-1 expression, and APOA-1DNA methylation in patients with HCC.

**Methods:** Data mining was performed using clinical and survival data from the Cancer Genome Atlas (TCGA) and Oncomine databases. The serum concentration of APOA-1 was measured in 316 patients with HCC and 100 healthy individuals at Renmin Hospital of Wuhan University, and the intact clinical information was reviewed and determined using univariate and multivariate Cox hazard models.

**Results:** Bioinformatic analysis revealed that APOA-1 mRNA was present at lower levels in the serum of patients with HCC than in that of healthy individuals, and there was a strong negative correlation between levels of APOA-1 mRNA and APOA-1 DNA methylation. High expression of APOA-1 transcription correlated with better overall survival (p = 0.003), and APOA-1 hypermethylation correlated with progress-free survival (p = 0.045) in HCC sufferers. Next, the clinical data analysis demonstrated that APOA-1 protein levels in the serum were significantly lower in patients with HCC than in healthy controls. Furthermore, the expression of APOA-1 was significantly associated with some significant clinical indexes, and elevated APOA-1 expression was significantly associated with favorable (OS; HR:1.693, 95% CI: 1.194–2.401, p = 0.003) and better progression-free survival (PFS; HR = 1.33, 95% CI = 1.194–2.401, p = 0.045). Finally, enrichment analysis suggested that co-expressed genes of APOA-1 were involved in lipoprotein metabolism and FOXA2/3 transcription factor networks.

**Conclusion:** APOA-1 mRNA expression is negatively regulated by DNA methylation in HCC. Low expression of APOA-1 might be a potential risk biomarker to predict survival in patients with HCC.

## Introduction

Hepatocellular carcinoma (HCC) is the largest primary liver cancer subtype (80–90%) and one of the most common clinical malignancies (Villanueva, 2019). It has the third-highest cancer mortality rate among all malignant tumors and has limited therapeutic options (Sharma, 2020). In the past few decades, although the diagnostic methods and treatment schedules of HCC have progressed, the long-term survival of patients has not significantly improved owing to delayed diagnosis, easy metastasis, and common relapse (Tellapuri et al., 2018; Pinto Marques et al., 2020). Therefore, the search for a novel and reliable biomarker for predicting the prognosis of patients with HCC is of great importance.

Recent studies have pointed out that abnormal lipid metabolism plays an important role in tumor progression (Cine et al., 2014; Guo et al., 2019). Apolipoprotein A1 (APOA-1) is the main protein component of high-density lipoproteins; it has been reported to be involved in numerous biological processes, inhibiting the formation of tumor blood vessels and inducing tumor immune microenvironment to prevent malignant tumor development (Borgquist et al., 2016). In this regard, Gao et al. suggested that APOA-1 may be used as a potential therapeutic target for cancer treatment (Gao et al., 2011; Zamanian-Daryoush et al., 2013).

With the development of high-throughput sequencing technology, epigenetic regulation has become a research focus in recent years. One of the epigenetic regulators, DNA methylation, may disrupt the regulation of specific promoters in cancer and play a vital role in tumorigenesis (Skvortsova et al., 2019). Additionally, aberrant DNA methylation has been perceived as a biomarker of HCC (Cancer Genome Atlas Research Network et al., 2017; C.; Zhang et al., 2016). Long et al.(Long et al., 2019) established a diagnostic, prognostic, and recurrence model for distinguishing HCC, including two DNA methylation-driven genes. Hence, identification of the correlation between mRNA expression and DNA methylation will provide crucial insights into the molecular mechanisms of HCC and may offer novel research directions for individualized treatment of patients with HCC.

Several studies have explored the association between APOA-1 and survival, but the available data on APOA-1 in HCC are limited. For example, MA et al. (Ma et al., 2016) found that the concentration of serum APOA-1 is associated with tumor-free survival and overall survival (OS) in HCC after curative resection. However, no study has specifically investigated the correlation between APOA-1 transcription and APOA-1 DNA methylation. Hence, whether APOA-1 DNA methylation can affect the prognosis of patients with HCC is currently unknown. Thus, in this study based on data mining, we first attempted to investigate the association between the differential expression of APOA-1 transcription between HCC specimens and correspondingly normal tissues across all available datasets. We then also explored the prognostic role of APOA-1 mRNA and APOA-1 DNA methylation in patients with HCC in a public database. Finally, we retrospectively collected clinicopathological data of 316 patients with HCC undergoing surgical treatment and 100 healthy controls from the Renmin Hospital of Wuhan University; this data was used to explore the importance of serum APOA-1 protein in evaluating the prognosis of patients with HCC to provide new indicators for the diagnosis and prognostic assessment of HCC.

## Materials and Methods

### Bioinformatics Analysis in Public Databases

To evaluate the level of APOA-1 expression across all human cancers, we examined the transcription level of APOA-1 from the Oncomine database (Tian et al., 2020) (https://www.oncomine.org/resource/login.html). Expression profiles of APOA-1 mRNA in different cancer types were further verified using the Tumor IMune Estimation Resource (TIMER) (T. Li et al., 2017). To further explore the correlation between APOA-1 mRNA expression, APOA-1 DNA methylation and OS/PFS, the corresponding clinical information of 365 patients with HCC was examined by data mining in TCGA (up to September 1, 2020) using the UCSC Xena Browser (https://xenabrowser.net) (Liang et al., 2020) (T. Liang et al., 2020). Besides, the GSE54503 microarray dataset, in current research were acquired from the Gene Expression Omnibus (GEO) database (Y. Li et al., 2018), including gene DNA-methylation profiles (66 HCC samples and 66 nontumor samples) to detect the APOA-1 DNA methylation statues in HCC tumor tissues and normal sample.

We utilized the Co-expression module from Cbioportal website (http://www.cbioportal.org/) to identify the most correlated gens with APOA-1, and we also adopted STRING (https://www.string-db.org/) to search for the Protein-Protein Interaction (PPI) network of APOA-1 in HCC. FunRich software (Benito-Martin and Peinado, 2015) was employed to take functional enrichment analysis and investigate the mechanisms related to APOA-1 expression in HCC tissues. We considered that the differences were statistically significant in *p*-value < 0.05 and false discovery rate <25%.

In this analysis, the median value of APOA-1 transcription in TCGA was used to divided HCC patients into low and high APOA-1 groups. Similarly, the median value of APOA-1 DNA methylation in TCGA was set to divided HCC patients into hypomethylation and hypomethylation groups. All data were analyzed according to relevant regulations and guidelines.

### Collection of Serum APOA-1 Protein of Patients with HCC and Healthy Control Individuals.

The intact clinicopathological parameters of 316 patients with HCC and 100 healthy individuals from Renmin Hospital of Wuhan University from January 2015 to January 2017 were retrospectively collected and reviewed. The criteria used to recruit patients with HCC were: 1) patients without radiotherapy, chemotherapy, or targeted therapy before enrollment; 2) patients diagnosed with HCC after histological examination; 3) patient complete clinical data available; 4) patient informed consent provided.

Individuals were excluded from the study based on 1) patients combined with other malignancies; 2) patients with metabolic diseases (history of diabetes, dyslipidemia, hyperuricemia, Non-alcoholic fatty liver disease and related Lipid modifying drug use); and 3) patients with missing information. In addition, all healthy individuals were recruited from the Health Examination Center; all had received complete health checks, and none of them had a history of malignant tumors. The study was approved by the Ethics Committee of Renmin Hospital of Wuhan University, Hubei Province (No. WDRY2019-K104). All participants provided written informed consent after the study protocol was fully explained, and this study was performed in accordance with the Helsinki Declaration.

### Follow-Up

Survival analysis started from the day of surgery and consisted of the survival time. The patients were approached by telephone and outpatient follow-up. To record the survival status of the patients, follow-ups were conducted every 3 months within half a year after the operation and then every 6 months. OS was defined as the time from diagnosis to death due to any reason or the last follow-up. Progression-free survival (PFS) was calculated as the interval between the first diagnosis and the date of recurrence. The last follow-up date was December 31, 2019.

### Statistical Analysis

Statistical analyses were performed using SPSS version 21.0 (IBM Corporation) and GraphPad Prism 5 (San Diego, CA) software. The experimental values for continuous variables are expressed as the mean ± standard error of the mean. The chi-squared test, Fisher’s exact probability test, and Student’s t-test were used to determine the significance of the differences in the data between groups. Receiver operating characteristic (ROC) analysis was used to assess the diagnostic value of APOA-1 mRNA in differentiating HCC tissues from normal tissues. Survival analyses were performed using Kaplan–Meier curves and the log-rank test. Univariate and multivariate Cox regression proportional hazards analyses were used to assess significant prognostic factors among the clinicopathological features. All *p*-values were determined from two-tailed tests. Differences with a *p*-value < 0.05 were considered to be statistically significant.

## Results

### APOA-1 Expression in HCC and Normal Tissues

**FIGURE 1:**
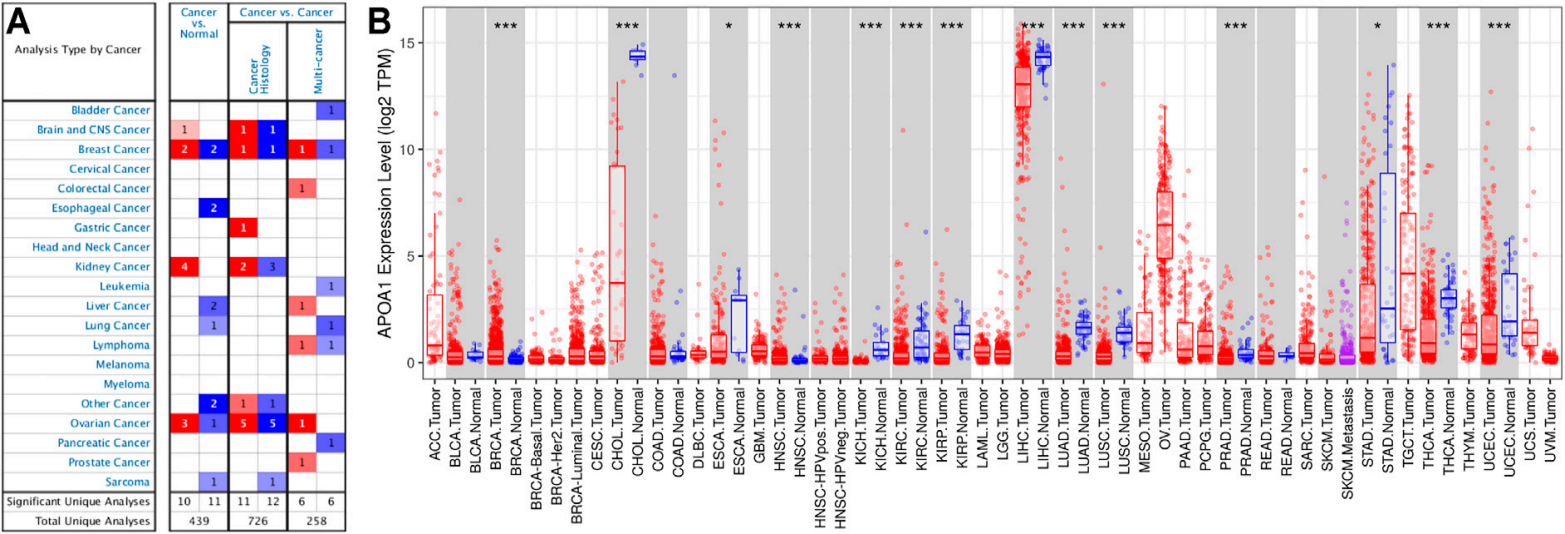
Expression of APOA-1 in human cancers. **(A)** Disease summary for APOA-1. Red: high expression, blue: low expression. The threshold was set as follows: *p*-value < 0.05, fold change: 2, gene rank: 10%; **(B)** Bar plot of APOA-1 expression profile in various cancers and normal specimens. All data were derived from the Oncomine database and the Tumor IMune Estimation Resource (TIMER).

The differential expression of APOA-1 in various types of tumors and normal individuals was explored using the Oncmine database. APOA-1 was lowly expressed in HCC, lung cancer, esophageal cancer, and sarcoma and highly expressed in breast, kidney, and ovarian cancers (Figure 1A). Moreover, this differential expression of APOA-1 was also verified with data from the TIMER database (Figure 1B). The results revealed that the expression of APOA-1 is varied with different types of cancer.

**FIGURE 2:**
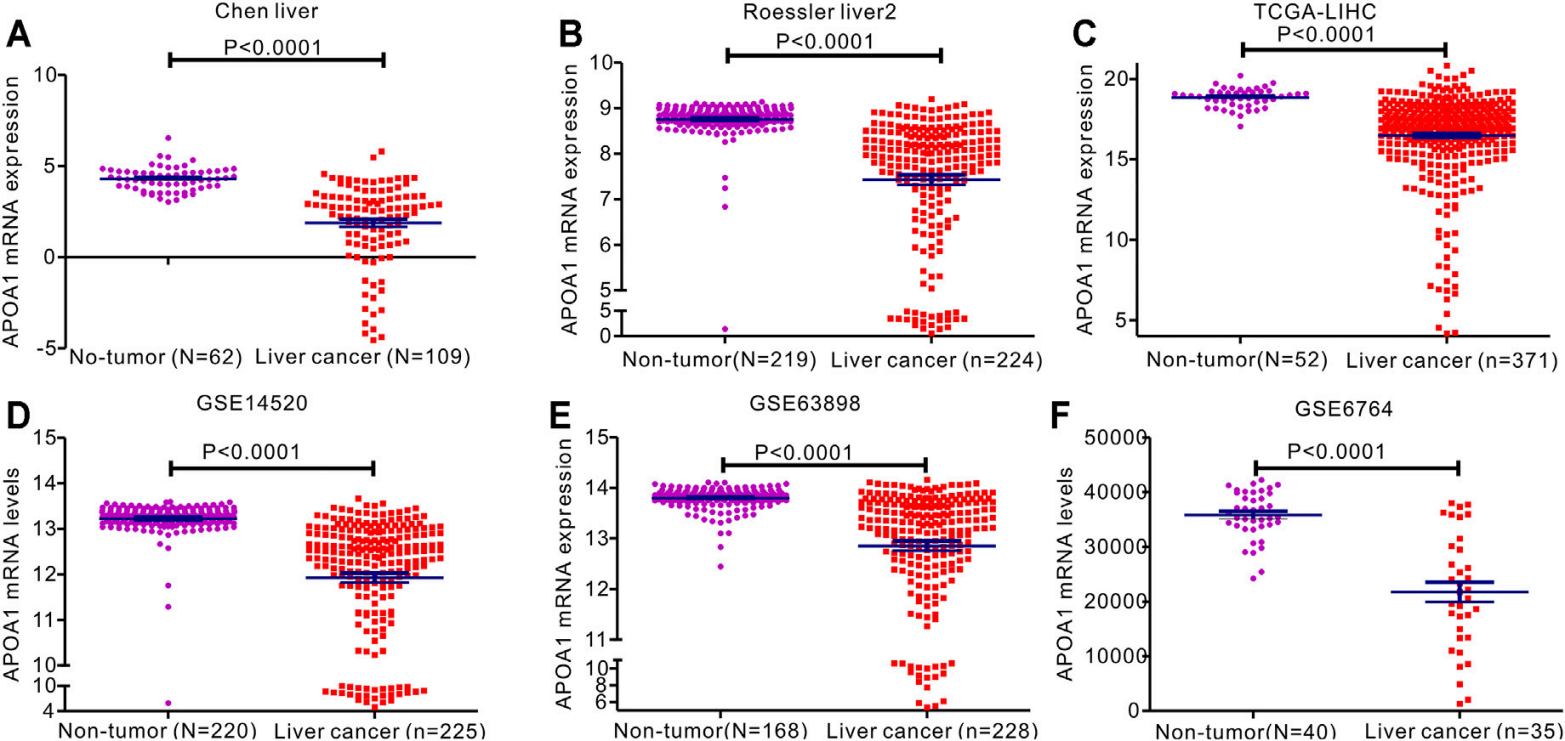
Expression of APOA-1mRNA expression in patients with HCC. Levels of serum APOA-1 are higher in healthy controls than in patients with HCC. Differential expression of APOA-1 in **(A)** Chen liver; **(B)** Roessler liver 2; **(C)** TCGA-LIHC; **(D)** GSE14520; **(E)** GSE63898; and **(F)** GSE6764.

The expression of APOA-1 in HCC was further analyzed in specific datasets. Next, we used related HCC datasets from the Oncomine and GEO databases to explore APOA-1 transcription in HCC and corresponding normal tissues. As shown in Figure 2, APOA-1 mRNA levels were significantly downregulated in HCC tissues than in normal tissues. Significant differences were observed (*p* < 0.001) in the Chen liver (Figure 2A), Roessier liver 2 (Figure2B), TCGA-LIHC (Figure 2C), GSE14520 (Figure 2D), GSE63898 (Figure 2E), and GSE6764 (Figure 2F) HCC datasets.

**FIGURE 3:**
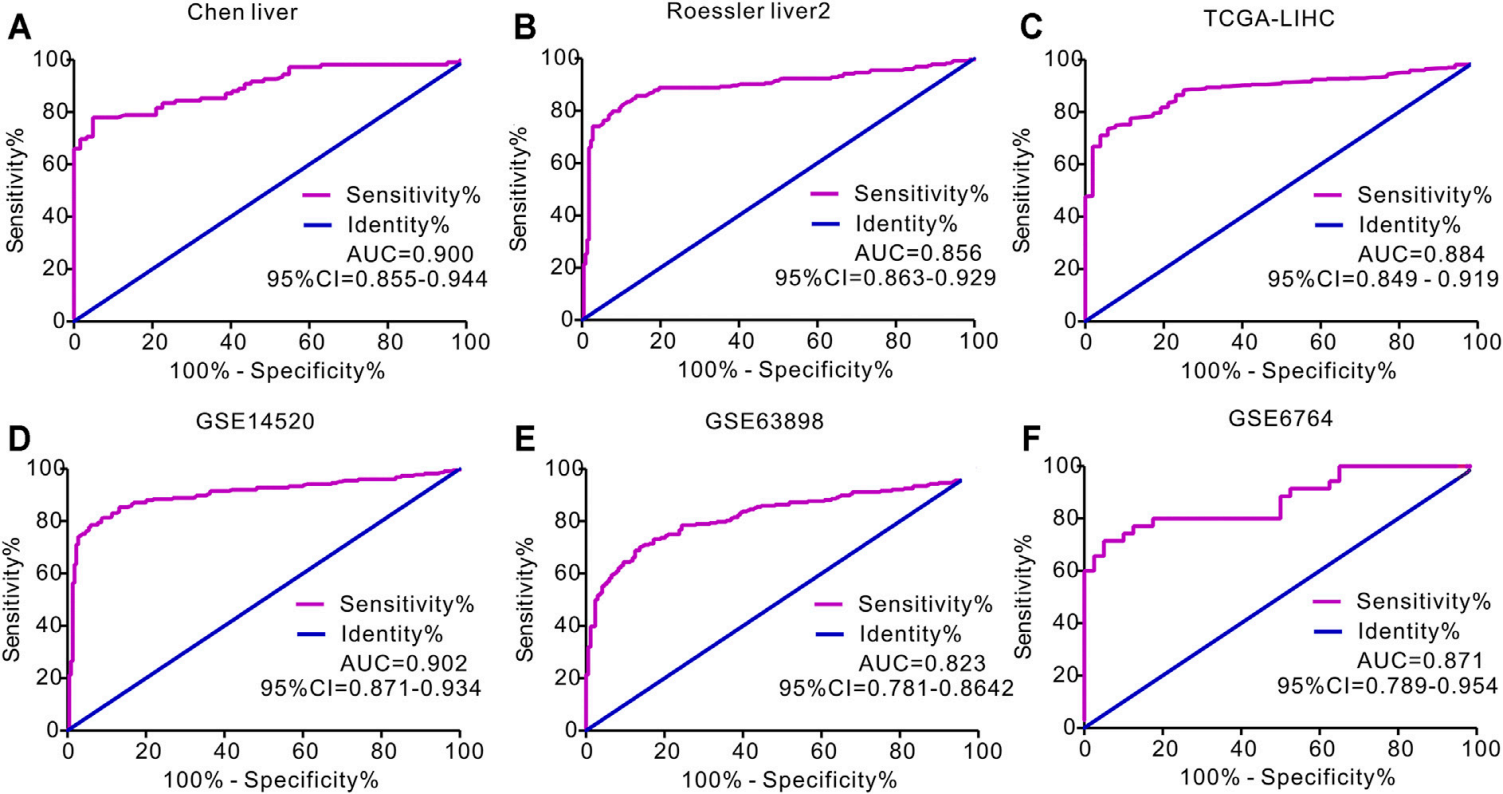
Receiver operating characteristics (ROC) curve for APOA-1 mRNA expression in predicting HCC. ROC curve of APOA-1 in **(A)** Chen liver, **(B)** Roessler liver 2, **(C)** TCGA-LIHC, **(D)** GSE14520, **(E)** GSE63898, and **(F)** GSE6764.

We then used ROC curves to determine the ability of circulating APOA-1 protein to discriminate between individuals with and without HCC. The result indicated that circulating APOA-1 protein was a good discriminator with good diagnostic efficiency (Figures 3A–F). Serum APOA-1 protein showed the highest diagnostic potential to discriminate HCC tissues from normal tissues in the GSE14520 dataset, as reflected by an area under the curve (AUC) of 0.902 (Figure 3D).

### APOA-1 mRNA Transcription Is Negatively Regulated by DNA Methylation

**FIGURE 4:**
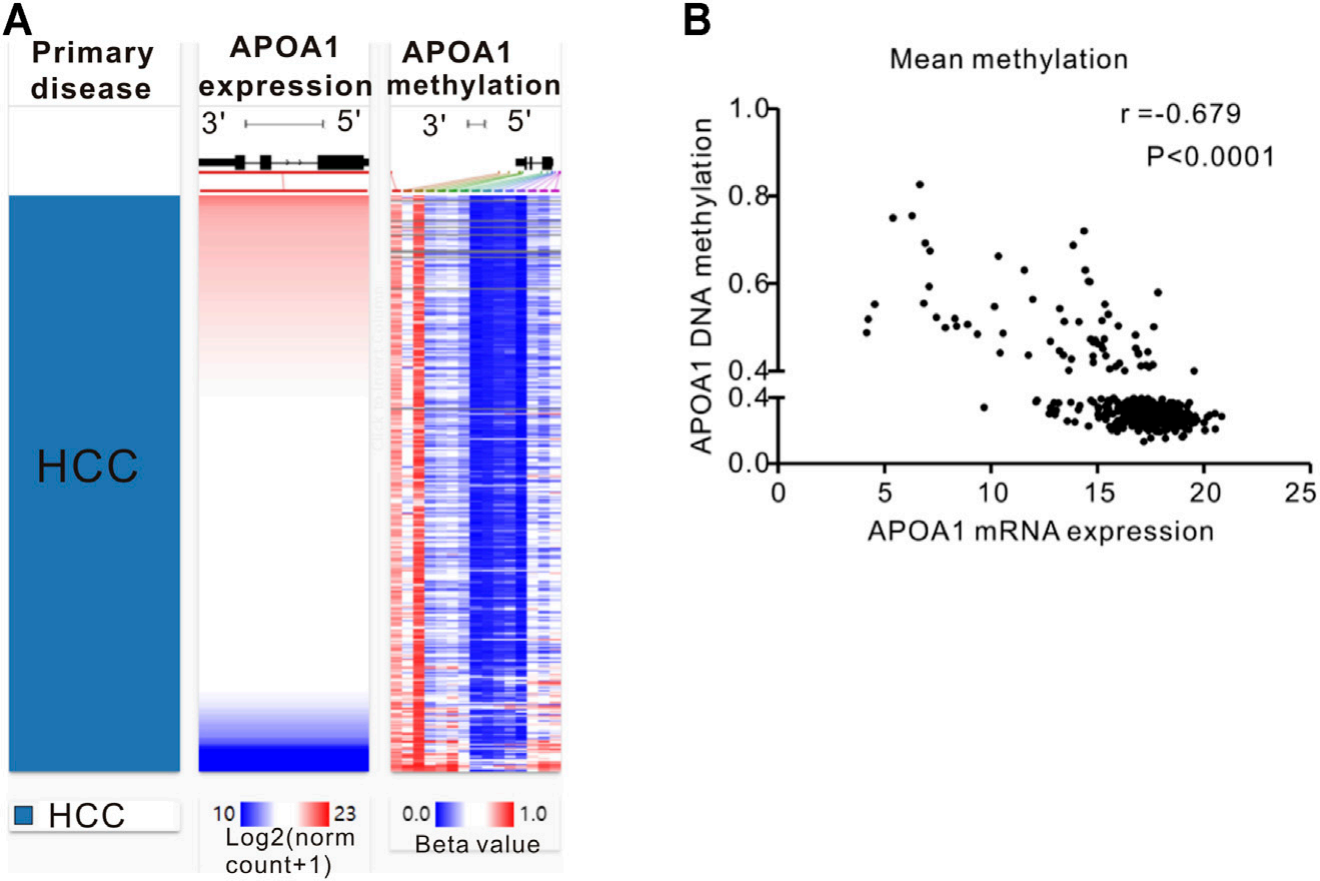
The epigenetic mechanism underlying aberrant expression of APOA-1 in HCC revealed by bioinformatic analysis. **(A)** Heatmap of the association between expression of APOA-1 mRNA and the methylation of APOA-1 DNA CpG sites; **(B)** a negative correlation is observed between APOA-1 mRNA and APOA-1 DNA methylation.

**FIGURE 5:**
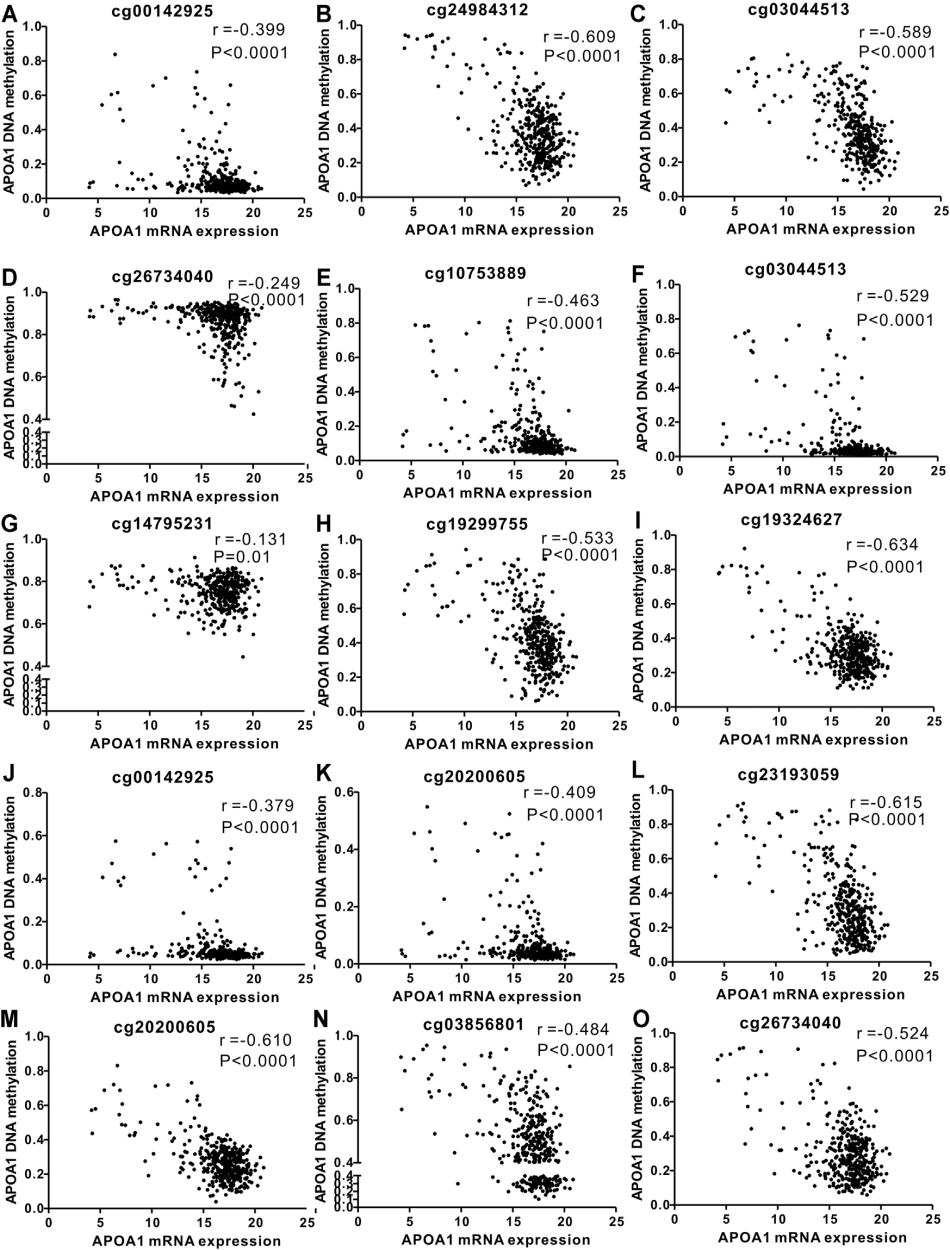
Correlation analysis of the relationship between APOA-1 mRNA expression and methylation of APOA-1 DNA CpG sites in TCGA dataset. **(A)** cg00142925; **(B)** cg24984312; **(C)** cg03044513; **(D)** cg26734040; **(E)** cg10753889; **(F)** cg03044513; **(G)** cg14795231; **(H)** cg19299755; **(I)** cg19324627; **(J)** cg00142925; **(K)** cg20200605; **(L)** cg23193059; **(M)** cg20200605; **(N)** cg03856801; and **(O)** cg26734040.

A comparable heatmap (Figure 4A) was generated using the UCSC Xena website, which included APOA-1 mRNA expression and APOA-1 DNA methylation in the TCGA-LIHC dataset. The results demonstrated that the expression levels of APOA-1 were significantly related to DNA methylation. As illustrated in Figure 4, APOA-1 mRNA expression was significantly negatively regulated by DNA methylation (Spearman, r = −0.679, *p* < 0.0001). In addition, the association between APOA-1 mRNA expression and APOA-1 DNA methylation levels of specific CpG sites was also studied using Spearman correlation analysis (Supplementary Table S1). Both the heatmap and the regression analysis showed that APOA-1 mRNA expression was negatively correlated with the 15 CpG sites of APOA-1 DNA methylation (Figure 5). Additionally, we also mined a HCC dataset related to DNA methylation (GSE54503). As illustrated in Supplementary Figure S1A, we found that average methylation levels of APOA-1 were significantly higher in healthy individuals than in patients with HCC from TCGA dataset (*p* < 0.005). We then analyzed the GSE54503 database, and the results show average methylation levels of APOA-1 were also significantly higher in healthy individuals than in patients with HCC (Supplementary Figure S1B,C).

### Prognostic Value of APOA-1 mRNA and DNA Methylation in Patients With HCC in TCGA Database

**FIGURE 6:**
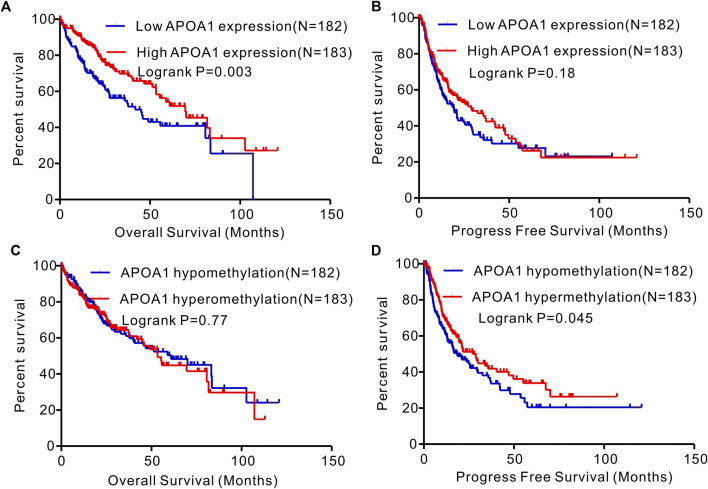
Kaplan–Meier curves of OS and PFS in patients with HCC in the TCGA database. **(A)** High expression APOA-1 is associated with better OS in patients with HCC; **(B)** but not associated with PFS. **(C)**APOA-1 hypermethylation is not correlated with OS in patients with HCC; **(D)** but correlated with favorable PFS.

The associations between APOA-1 mRNA expression and clinical parameters among the 365 patients with HCC were statistically analyzed. As demonstrated in Supplementary Table S2, high APOA-1 mRNA levels were significantly correlated with advanced race (*p* = 0.010), α-fetoprotein (AFP) (*p* < 0.001), BMI (*p* = 0.033), and methylation status (*p* < 0.001), whereas no correlations were observed with age (*p* = 0.238), gender (*p* = 0.316), G stage (*p* = 0.110), or M stage (*p* = 0.841). The Kaplan–Meier curves were then used to explore the prognostic value of APOA-1 mRNA in the 365 patients with HCC. As illustrated in Figure 6, high APOA-1 mRNA expression was significantly associated with good OS (*p* = 0.003, Figure 6A). However, PFS showed no correlation with the level of APOA-1 mRNA expression (*p* = 0.18, Figure 6B). Kaplan-Meier survival analysis was used to descriptively show the association between APOA-1 mRNA and OS based on the GSE14520 dataset as validation. The results also indicate that the low APOA-1 group patients have a worse prognosis (Supplementary Figure S2).

Additionally, based on the 365 cases of HCC with corporate clinical information and APOA-1 methylation data, the chi-squared test or Fisher’s exact test was used to assess the correlations between APOA-1 DNA methylation and clinical features. As shown in Supplementary Table S2, the APOA-1 DNA methylation status was influenced by race (*p* = 0.034), BMI (*p* < 0.001), and APOA-1 mRNA expression levels (*p* < 0.001). As exhibited in the Kaplan–Meier curves, the OS showed no correlation with low or high APOA-1 DNA methylation (*p* = 0.77, Figure 6C), while APOA-1 DNA hypermethylation was significantly associated with favorable PFS (*p* = 0.045, Figure 6D).

Multivariate Cox regression analyses were performed to identify independent risk factors for the prognosis of patients with HCC. Table 1 demonstrates that the serum level of APOA-1 was an independent prognostic factor for OS among patients with HCC (HR = 0.594, 95% CI = 0.419–0.842, *p* = 0.007). T stage (HR = 22.649,95% CI = 1.605–3.022, *p* < 0.001) and TNM staging (HR = 2.222, 95% CI = 1.603–3.081, *p* < 0.001) were independent prognostic factors for PFS among patients with HCC.

**TABLE 1:** Multivariate Cox regression analyses of independent prognostic factors for overall survival and Progression-free survival in patients with HCC from TCGA cohort.

Variables		OS		PFS	
		HR (95% CI)	P	HR (95% CI)	P
Age	≤55	1.173 (0.807–1.706)	0.403		
	>55			0.968 (0.709–1.32)	0.836
G stage	G1+G2				
	G3+G4	1.114 (0.776–1.599)	0.559	1.166 (0.859–1.582)	0.325
M stage	M0				
	M1+Mx	1.176 (1.211–2.519)	**0.003**	1.258 (0.912–1.734)	0.162
N stage	N0				
	N1+Nx	1.519 (1.052–2.193)	**0.026**	1.222 (0.889–1.681)	0.216
T stage	T1+T2				
	T3+T4	2.483 (1.746–3.531)	**<0.001**	22.649 (1.605–3.022)	**<0.001**
Race	White				
	Asia	0.911 (0.395–2.100)	0.828	0.77 (0.566–1.048)	0.097
	Black or African American	0.673 (0.286–1.583)	0.364	0.583 (0.256–1.33)	0.200
	American Indian or Alaska Native	—			
Radiation Therapy	No				
	Yes	0.929 (0.295–2.926)	0.900	1.557 (0.688–3.524)	0.288
Sex	female				
	male	0.805 (0.565–1.148)	0.232	0.939 (0.688–1.283)	0.694
AFP ng/mL	≤400				
	>400	0.787 (0.495–1.252)	0.312	0.956 (0.647–1.413)	0.823
BMI	<18.5	1.296 (0.793–2.119)	0.301	1.227 (0.788–1.909)	0.365
	18.5–24	0.799 (0.544–1.174)	0.253		
	>24			0.903 (0.654–1.246)	0.533
APOA-1	≤17.12				
	>17.12	0.594 (0.419–0.842)	**0.003**	0.818 (0.609–1.098)	0.181
APOA-1 methylation	≤0.3147				
	>0.3147	1.053 (0.745–1.488)	0.769	0.752 (0.559–1.011)	0.059
TNM staging	I + II				
	III + IV	2.373 (1.639–3.435)	**<0.001**	2.222 (1.603–3.081)	**<0.001**

### Correlation Between Serum APOA-1 Protein and Clinical Features

**FIGURE 7:**
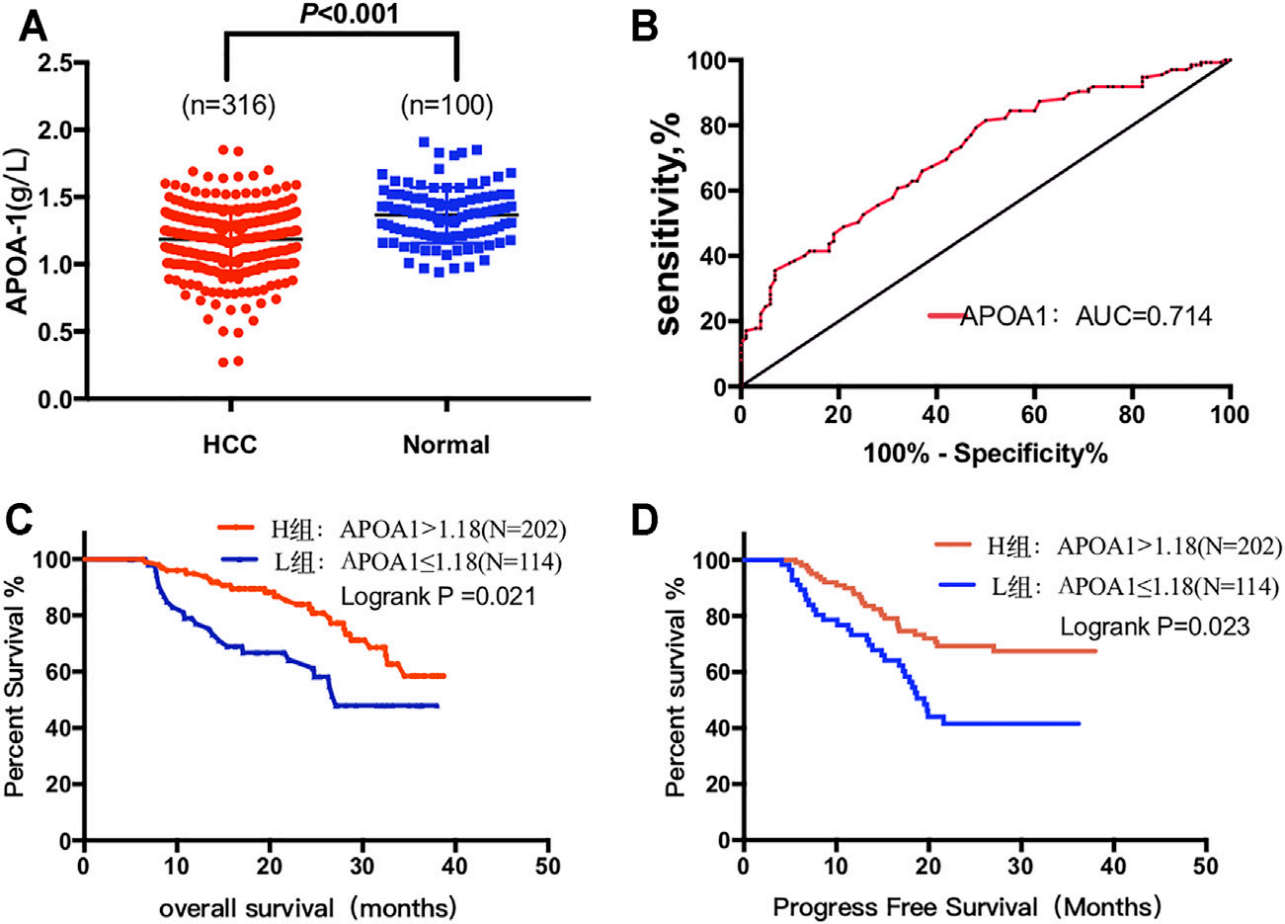
Circulating serum APOA-1 protein in patients with HCC. **(A)** serum APOA-1 protein is higher in healthy controls than in HCC; **(B)** AUC for APOA-1. High expression APOA-1 is associated with better **(C)** OS and **(D)** PFS in 316 patients with HCC.

As shown in Figure 7A, serum APOA-1 levels of healthy individuals was significantly higher than that in patients with HCC (1.46 ± 0.21 vs. 1.20 ± 0.21 g/L, *p* < 0.001). Next, ROC analysis was used to evaluate the measurement accuracy of serum APOA-1 in differentiating patients with HCC from healthy controls. The AUC of serum APOA-1 for identifying patients with HCC from healthy controls was 0.714, the cutoff value of APOA-1 was 1.18 ug/mL, and the sensitivity and specificity were 50.5 and 81.3%, respectively (Figure 7B).

Furthermore, the correlation between serum APOA-1 protein levels and clinical features has been detected and summarized in Table 2. Specifically, serum APOA-1 levels were significantly correlated with differences in hs-CRP (*p* = 0.003), HBsAg (*p* < 0.001), tumor number (*p* < 0.001), Child-Pugh score (*p* = 0.027), and BCLC stage (*p* = 0.041). However, no statistical significance was observed with age (*p* = 0.580), gender (*p* = 0.359), or AFP (*p* = 0.732).

**TABLE 2:** Correlation between serum APOA-1 levels and clinicopathologic characteristics of 316 patients with HCC.

Clinical characteristics		Low APOA-1 (≤1.18, N = 114)	High APOA-1 (>1.18, N = 202)	P
Age(years)	≤50	34 (29.8%)	52 (25.7%)	0.580
	>50	80 (70.2%)	150 (74.3%)	
Gender	Male	100 (87.7%)	166 (82.2%)	0.359
AFP, ng/mL	≤400	72 (63.2%)	122 (60.4%)	0.732
	>400	42 (36.8%)	80 (39.6%)	
ALT, U/L	≤75	98 (86.0%)	180 (89.1%)	0.560
	>75	16 (14.0%)	22 (10.9%)	
Fn, mg/L	≤280	102 (89.5%)	170 (84.2%)	0.354
	>280	12 (10.5%)	32 (15.8%)	
hs-CRP, mg/L	≤10	42 (36.8%)	109 (54.0%)	**0.003**
	>10	72 (63.2%)	93 (46.0%)	
APOB	≤0.84	66 (57.9%)	98 (48.5%)	0.257
	>0.84	48 (42.1%)	104 (51.5%)	
NLR	≤4.2	78 (68.4%)	154 (76.2%)	0.466
	>4.2	36 (31.6%)	48 (23.8%)	
HBsAg	Negative	30 (26.3%)	152 (75.2%)	**<0.001**
	Positive	84 (73.7%)	50 (24.8%)	
Liver cirrhosis	No	54 (47.4%)	92 (45.5%)	0.755
	Yes	60 (52.6%)	110 (54.5%)	
Tumor size,cm	≤5	50 (43.9%)	96 (47.5%)	0.632
	>5	64 (56.1%)	106 (52.5%)	
No.of tumors	Single	62 (54.4%)	166 (82.2%)	**<0.001**
	Multiple	52 (45.6%)	36 (17.8%)	
Satellite lesion	No	84 (73.7%)	172 (85.1%)	0.078
	Yes	30 (26.3%)	30 (14.9%)	
Vascular invasion	No	62 (54.4%)	130 (64.4%)	0.218
	Yes	52 (45.6%)	72 (35.6%)	
Tumor differentiation	Well	32 (28.1%)	66 (32.7%)	0.315
	Moderately	54 (47.4%)	106 (52.5%)	
	Poorly	28 (24.6%)	30 (14.9%)	
Child-Pugh score	A	76 (66.7%)	166 (82.2%)	**0.027**
	B	38 (33.3%)	36 (17.8%)	
BCLC stage	0 + A	72 (63.2%)	158 (78.2%)	**0.041**
	B + C	42 (36.8%)	44 (21.8%)	

### APOA-1 Was an Independent Prognostic Marker for HCC

We further performed survival analysis to explore the correlation between serum APOA-1 levels and survival outcomes in the 316 of patients with HCC. Kaplan–Meier survival curves were constructed to determine the prognostic significance of circulating APOA-1 protein levels in HCC. High serum APOA-1 protein levels were significantly associated with good OS (*p* = 0.021, Figure 7C) and PFS (*p* = 0.023, Figure 7D).

Intact clinical information was included in the univariate and multivariate analyses to detect the association between serum APOA-1 protein levels and HCC patient prognosis. Multivariate analyses were performed to identify the factors that were identified in the univariate analyses. As shown in Table 3, hs-CRP (HR = 1.698, 95% CI = 1.010–2.853, *p* = 0.046), BCLC stage (HR = 1.285, 95% CI = 1.132–1.770, *p* = 0.023), and APOA-1 (HR = 0.831, 95% CI = 0.482–0.940, *p* < 0.001) were identified as significant independent predictors of OS in patients with HCC. Besides, hs-CRP (HR = 1.632, 95% CI = 1.353–2.782, *p* = 0.018), NLR (HR = 2.546, 95% CI = 1.415–4.579, *p* = 0.002), tumor size (HR = 1.828, 95% CI = 1.059–3.156, *p* = 0.030), tumor differentiation (HR = 1.605, 95% CI = 1.059–2.433, *p* = 0.026) and APOA-1 (HR = 0.682, 95% CI = 0.390–0.912, *p* < 0.001) were independent prognostic indicators of PFS (Table 4).

**TABLE 3:** Univariate and multivariate cox hazards analysis for Overall survival in 316 patients with HCC.

Variables	Univariate analysis		Multivariate analysis	
	HR (95% CI)	P	HR (95%CI)	P
Gender	0.783 (0.436–1.409)	0.415		
Male vs. Female				
Age(years)	1.036 (0.650–1.653)	0.881		
≤50 vs.>50				
AFP, ng/mL	1.551 (1.110–2.164)	**0.008**	0.961 (0.469–1.970)	0.961
≤400 vs.>400				
ALT, U/L	1.203 (0.681–2.132)	0.545		
≤75 vs.>75				
Fn	1.591 (0.851–2.975)	0.146		
≤280 vs.>280				
hs-CRP	1.699 (1.173–2.242)	**0.023**	1.698 (1.010–2.853)	**0.046**
≤10 vs.>10				
APOB	1.358 (0.852–2.165)	0.199		
≤0.84 vs.>0.84				
NLR	1.571 (1.272–2.049)	**0.008**	0.567 (0.297–1.079)	0.567
≤4.2 vs.>4.2				
HBsAg	1.346 (0.832–2.177)	0.226		
Negative vs. Positive				
Tumor size(cm)	1.921 (1.559–2.461)	**<0.001**	0.811 (0.482–1.366)	0.431
≤5 vs.>5				
Tumor number	1.563 (1.243–2.568)	**<0.001**	1.254 (0.605–2.599)	0.542
single vs. Multiple				
Satellite lesion	1.681 (1.482–2.413)	**0.002**	1.283 (0.922–1.763)	0.140
No vs. Yes				
Vascular invasion	1.553 (0.814–2.903)	0.129		
No vs.Yes				
Tumor differentiation	1.112 (1.799–1.547)	**0.043**	1.011 (0.711–1.437)	0.952
Well vs. Moderately vs. Poorly				
Child-Pugh score	1.791 (1.070–2.998)	**0.027**	1.592 (0.805–3.150)	0.181
A vs. B				
BCLC stage	1.306 (1.020–1.672)	**0.034**	1.285 (1.132–1.770)	**0.023**
0+A vs. B + C				
APOA-1	0.732 (0.452–0.985)	**0.004**	0.831 (0.482–0.940)	**<0.001**
≤1.18 vs.>1.18				

**TABLE 4:** Univariate and multivariate cox hazards analysis for Progression-free survival in 316 patients with HCC.

Variables	Univariate analysis		Multivariate analysis	
	HR (95% CI)	P	HR (95%CI)	P
Gender	0.705 (0.365–1.361)	0.298		
Male vs. Female				
Age(years)	0.775**(**0.458–1.312)	0.343		
≤50 vs.>50				
AFP, ng/mL	1.502**(**1.312–1.163)	**0.015**	1.355 (0.621–2.955)	0.446
≤400 vs.>400				
ALT, U/L	1.156 (0.729–1.836)	0.545		
≤75 vs.>75				
Fn	1.064 (0.522–2.169)	0.865		
≤280 vs.>280				
hs-CRP	1.974 (1.152–3.384)	**0.013**	1.632 (1.353–2.782)	**0.018**
≤10 vs.>10				
APOB	1.052 (0.626–1.769)	0.849		
≤0.84 vs.>0.84				
NLR	3.250 (1.912–5.522)	**<0.001**	2.546 (1.415–4.579)	**0.002**
≤4.2 vs.>4.2				
HBsAg	1.179 (0.692–2.008)	0.544		
Negative vs. Positive				
Tumor size(cm)	1.911 (1.121–3.259)	**0.017**	1.828 (1.059–3.156)	**0.030**
≤5 vs.>5				
Tumor number	0.553 (0.221–1.385)	0.206		
Single vs. Multiple				
Satellite lesion	0.552 (0.220–1.383)	0.205		
No vs. Yes				
Vascular invasion	0.774 (0.485–1.142)	0.189		
No vs.Yes				
Tumor differentiation	1.704 (1.172–2.479)	**0.005**	1.605 (1.059–2.433)	**0.026**
Well vs. Moderately vs. Poorly				
Child-Pugh score	2.041 (1.191–3.500)	**0.009**	1.185 (0.574–2.446)	0.646
A vs. B				
BCLC stage	1.635 (1.260–2.122)	**<0.001**	1.217 (0.845–1.754)	0.291
0+A vs. B + C				
APOA-1	0.547 (0.323–0.926)	**0.004**	0.682 (0.390–0.912)	**<0.001**
≤1.18 vs.>1.18				

### Enrichment Analysis of Genes Co-expressed With APOA-1

**FIGURE 8:**
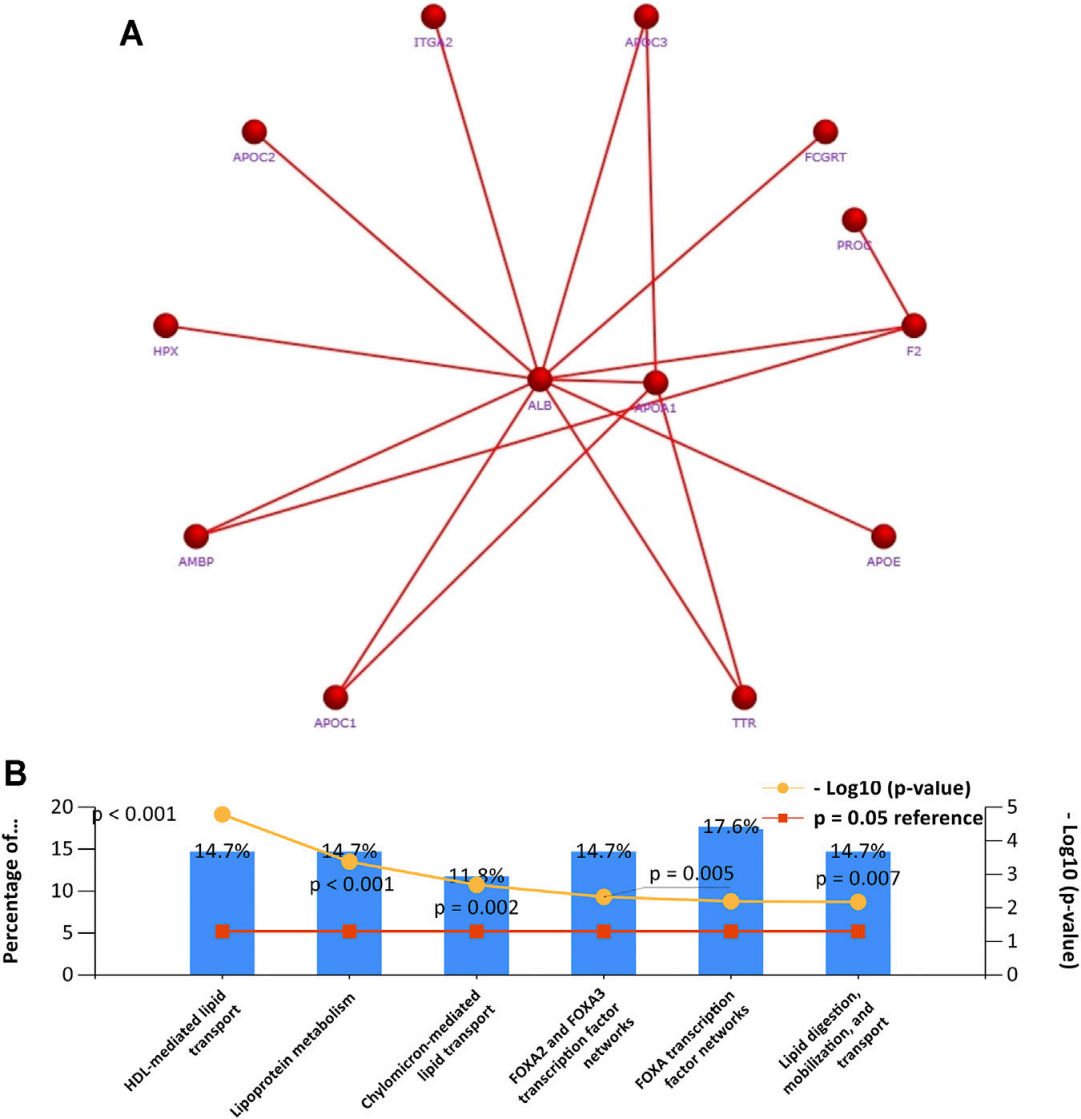
Gene set enrichment analysis of PCR-array. **(A)** The network of APOA-1 and its co-expressed genes in various cells; **(B)** gene set enrichment analysis of biological processes.

To further clarify the potential role of APOA-1 in HCC, we first selected genes that were co-expressed with APOA-1 in LIHC dataset. We then used STRING database to investigate the PPI network of APOA1 in HCC by setting *p*-value < 0.05 and false discovery rate <25% as threshold, and we found that APOA1 is the hub gene in HCC (Figure 8A). Finally, we applied enrichment analysis using FunRich software, implicating that co-expressed genes were significantly enriched in lipoprotein metabolism and FOXA2 and FOXA3 transcription factor networks (Figure 8B).

## Discussion

As one of the leading causes of cancer-related deaths, HCC is a significant public health burden (Sartoris et al., 2021). Therefore, it is of great importance to detect indicators for optimizing early diagnosis and improving the prognosis of HCC (Mustafa et al., 2013). In our study, according to the univariate and multivariate analysis results, the clinical value of traditional HCC diagnosis and prognostic indicators such as tumor size/number (Yim et al., 2016; Tzartzeva and Singal, 2018), degree of differentiation, and inflammatory factors (CRP, NLR) (Greten and Grivennikov, 2019; Huang et al., 2019; Chan et al., 2020; Rogovskii, 2020) have been verified. Moreover, public gene expression and DNA methylation data are easily available, presenting a valuable opportunity to study diseases at the gene level (Yang et al., 2020). Hence, we found that the lipid metabolism biomarker APOA-1 is closely related to the prognosis of patients with HCC. To our knowledge, this is the first study to combine data mining and clinicopathological variables to explore the relationships between APOA-1 mRNA/DNA-methylation/protein expression levels and prognosis.

In our study, we identified a potential tumor suppressor gene (TSG; APOA-1), which is commonly suppressed by DNA CpG methylation in HCC. Our study elucidated that both APOA-1 mRNA and DNA methylation are innovative prognostic biomarkers in patients with HCC using comprehensive database analysis. Furthermore, we analyzed the differential expression of APOA-1 at the protein level and found that serum APOA-1 protein levels were much lower in patients with HCC than in healthy control groups, which further indicated that APOA-1 might be a tumor suppressor protein in HCC. Taken together, our findings strongly suggest that APOA-1 is a critical tumor suppressor and APOA-1 hypomethylation could serve as an independent prognostic factor in HCC.

Recent studies have reported that abnormal lipid metabolism is closely correlated with the development of malignant tumors (Estirado et al., 2018). These results demonstrate that abnormal lipid metabolism increases the risk of colorectal cancer (X. Zhang et al., 2014), ovarian cancer (D. Zhang et al., 2020), breast cancer (Kumar et al., 2015), and other cancers. Jiang et al. (Jiang et al., 2016) found that low levels of high-density lipoprotein cholesterol (HDL-C) and cholesterol (CHO) are preoperative risk factors for PFS and OS in patients with HCC. The reduction in CHO levels was associated with a reduction in OS (*p* = 0.003) and a reduction in PFS (*p* = 0.012). These results clarify that abnormal lipid metabolism is involved in the progression of HCC. Currently, only two studies (Ma et al., 2016; M.; Mao et al., 2018) have investigated the prognostic role of APOA-1 mRNA in patients with HCC. These studies have ascertained that the low APOA-1 expression group has a higher risk of recurrence and a poor survival outcome which is consistent with the results of our study.

DNA methylation, one of the most common epigenetic processes, can alter gene expression without changing the DNA sequence (Skvortsova et al., 2019). CpG sites can accumulate in CpG islands, and the CpG islands in gene promoter regions are generally unmethylated under normal conditions. However, the amount of methylation in a CpG island can change because of gene regulation processes in the pathologic process (Greenberg and Bourc’his, 2019; Grosser and Metzler, 2020). In some types of malignant tumors, DNA hypermethylation in the promoter region can induce deregulated silencing of some TSGs (Jones, 2012). Previous studies have demonstrated that APOA-1 gene expression is related to DNA methylation. Wang et al. (Wang et al., 2016) revealed that APOA-1 mRNA expression was downregulated by hypermethylation of CpG islands for hepatitis B virus (HBV) infection, which may contribute to the development of cirrhosis, liver failure, and HCC. Unlike previous studies, our study revealed that APOA-1 DNA methylation levels in HCC tissues were lower than those in non-tumor tissues. This may be due to the complexity of the regulatory mechanism of mRNA expression. Thus, further research is needed to clarify this point. Furthermore, Kaplan–Meier survival curves demonstrated that the PFS time of patients with HCC with APOA-1 DNA hypermethylation was significantly longer than that of patients with APOA-1 DNA hypomethylation. Our study suggests that APOA-1 DNA hypermethylation may serve as a unique prognostic indicator for patients with HCC.

Additionally, APOA-1 protein, as a member of the apolipoprotein A1/A4/E family encoded by the APOA-1 gene, plays a vital role in the formation of lipoprotein complexes of low-density and high-density lipoproteins (Chyu and Shah, 2015). From this perspective, APOA-1 has been implicated in the progression and recurrence of many metabolic and cardiovascular diseases (Dufresne et al., 2019; C.; Li et al., 2019). Nonetheless, some investigators have increasingly turned their attention to the interaction between serum APOA-1 protein and cancer. Kim et al. (Kim et al., 2020) collected blood samples from 180 patients with pancreatic ductal adenocarcinoma (PDAC) and 573 healthy controls to determine whether APOA-1 is a new biomarker for early diagnosis of PDAC. Peng et al. (Peng et al., 2019) found that high-grade bladder cancer (BC) patients have significantly higher APOA-1 levels than do low-grade BC patients, indicating that circulating APOA-1 protein may be a novel biomarker for BC diagnosis and prognosis monitoring. In our study, we specifically observed that the expression of APOA-1 protein was significantly associated with hs-CRP, HBsAg, tumor number, Child-Pugh score, and BCLC stage. In addition, our results demonstrated that low APOA-1 levels were strongly correlated with inferior OS and PFS.

The exact mechanism of action of APOA-1 in tumorigenesis is unclear. Recent studies have reported that APOA-1 may be implicated in the involvement of tumor microenvironment, tumor growth, immune cells, and tumor cell proliferation (M. Mao et al., 2018). Moreover, Cristina et al. (Aguirre-Portoles et al., 2018) observed that APOA1 can reverse the malignant phenotypes displayed by cells overexpressing a cholesterol transport regulator (RCT). Thus, intracellular cholesterol metabolism and APOA-1 emerge as new relevant players in CRC progression to metastasis by modulating intracellular cholesterol metabolism. Mao et al. (J. Mao et al., 2014) demonstrated that APOA-1 overexpression is associated with the inhibition of COX-2 expression in hepatocytes. Besides, APOA-1 overexpression can reduce steatosis by decreasing reactive oxygen species (ROS) levels and suppressing COX-2-induced inflammation in hepatocytes. In addition, Fessler et al. found that APOA-1 can affect the response of immune cells to tumors by regulating the cholesterol content in membrane lipid rafts (Fessler and Parks, 2011). It can also inhibit the proliferation of tumor cells by promoting cell cycle arrest and promote apoptosis by regulating the mitogen-activated protein kinase pathway (Ma et al., 2016). At the genetic level, some studies (W. C. Liang et al., 2018; Liu et al., 2018) have shown that the Forkhead transcription family member FOXA2 plays a critical role in HCC progression and metastasis, which is consistent with our gene enrichment analysis results. This may be related to the mechanism of APOA-1 in tumorigenesis. Although further validation is required before the intact mechanism is clarified, the clinical use of APOA-1 has potential implications.

However, there were several limitations to our study. First, some recognized factors, such as tumor size, AFP, and vascular invasion, are not independent risk factors for OS and PFS in patients with HCC. This might be due to the relatively short follow-up time and the limitations of the small cohort size. It should be noted that most patients with HCC in China have been infected with HBV. However, abnormal levels of lipid metabolism variables may be associated with non-alcoholic fatty liver disease. Hence, a large-cohort, multi-center, long-term, and etiology-clear study including patients from different backgrounds is warranted in the future.

## Conclusion

Our study demonstrated that the expression of APOA-1 protein is significantly downregulated in HCC than in healthy individuals. APOA-1 DNA methylation, mRNA expression, and protein expression may act as vital predictors of the prognosis of patients with HCC undergoing surgical resection. APOA-1 hypermethylation is an independent protective factor for improved survival in patients with HCC. Additionally, further studies should aim to clarify the molecular mechanism that may facilitate the identification of new drug targets for HCC.

## Data Availability

The datasets presented in this study can be found in online repositories. The names of the repository/repositories and accession number(s) can be found in the article/Supplementary Material.

## Abbreviations

APOA-1, Apolipoprotein A1; AFP, α-fetoprotein; BCLC, Barcelona Clinic Liver Cancer; BP, biological process; BC, bladder cancer; CEA, carcinoembryonic antigen; CA199, Carbohydrate antigen199; CA125, Carbohydrate antigen125; CHO, cholesterol; HDL-C, high-density lipoprotein; HCC, hepatocellular carcinoma; HBV, Hepatitis B virus; OMAPK, mitogen-activated protein kinase; NAFLD, non-alcoholic fatty liver disease; OS, Overall survival; PDAC, pancreatic ductal adenocarcinoma; PFS,Progree-free survival; ROC, receiver operating characteristics; RCT, cholesterol transport regulator; ROS, reactive oxygen species; TSGs, tumor suppressor gene.
