# Supplementary material for: Low APOA-1 Expression in Hepatocellular Carcinoma Patients Is Associated With DNA Methylation and Poor Overall Survival
Source: Front Genet. 2021 Nov 1;12:760744. doi: 10.3389/fgene.2021.760744 (PMC8591198; doi:10.3389/fgene.2021.760744)
Supplement: Supplementary file 1 [file DataSheet1.docx]

**Supplementary materials**

**Table S1** Association between APOA-1 mRNA and specific APOA-1 DNA CpG site

| **APOA-1** | **Pearson** | **P value** | **Spearman** | **P** |
| --- | --- | --- | --- | --- |
| Methylation | -0.679 | <0.0001 | -0.529 | <0.0001 |
| cg14795231 | -0.131 | 0.01 | -0.044 | 0.4000 |
| cg25987102 | -0.487 | <0.0001 | -0.381 | <0.0001 |
| cg03856801 | -0.249 | <0.0001 | -0.275 | <0.0001 |
| cg26734040 | -0.524 | <0.0001 | -0.254 | <0.0001 |
| cg19324627 | -0.634 | <0.0001 | -0.339 | <0.0001 |
| cg03010018 | -0.609 | <0.0001 | -0.347 | <0.0001 |
| cg24984312 | -0.610 | <0.0001 | -0.407 | <0.0001 |
| cg20200605 | -0.409 | <0.0001 | -0.349 | <0.0001 |
| cg19360562 | -0.379 | <0.0001 | -0.352 | <0.0001 |
| cg00142925 | -0.399 | <0.0001 | -0.344 | <0.0001 |
| cg10753889 | -0.463 | <0.0001 | -0.421 | <0.0001 |
| cg13090478 | -0.529 | <0.0001 | -0.433 | <0.0001 |
| cg03044513 | -0.589 | <0.0001 | -0.597 | <0.0001 |
| cg23193059 | -0.616 | <0.0001 | -0.468 | <0.0001 |
| cg19299755 | -0.533 | <0.0001 | -0.468 | <0.0001 |

**Table S2** Correlation between APOA-1 mRNA expression/methylation and clinicopathologic features of 365 hepatocellular carcinoma patients in TCGA cohort

| **Variables** |  | **Low APOA-1 (N=182)** | **High APOA-1 (N=183)** | **P** | **Hypome thylation (N=182)** | **Hyperme thylation (N=183)** | **P** |
| --- | --- | --- | --- | --- | --- | --- | --- |
| Age | ≤55 | 56 | 67 | 0.238 | 57 | 66 | 0.337 |
|  | >55 | 126 | 116 |  | 125 | 117 |  |
| G stage | G1+G2 | 106 | 120 | 0.110 | 114 | 113 | 0.913 |
|  | G3+G4 | 74 | 59 |  | 66 | 67 |  |
| M stage | M0 | 132 | 131 | 0.841 | 133 | 130 | 0.664 |
|  | M1+Mx | 50 | 52 |  | 49 | 53 |  |
| N stage | N0 | 126 | 125 | 0.788 | 131 | 120 | 0.213 |
|  | N1+Nx | 55 | 58 |  | 51 | 62 |  |
| T stage | T1+T2 | 131 | 141 | 0.266 | 134 | 137 | 0.718 |
|  | T3+T4 | 51 | 42 |  | 48 | 45 |  |
| TNM staging | I+II | 118 | 136 | 0.138 | 127 | 127 | 0.856 |
|  | III+IV | 50 | 40 |  | 46 | 44 |  |
| Race | White | 98 | 82 | **0.010** | 80 | 100 | **0.034** |
|  | Asia | 69 | 87 |  | 89 | 67 |  |
|  | Black or African American | 15 | 5 |  | 6 | 11 |  |
|  | American Indian or Alaska Native | - | 3 |  | - | - |  |
| Radiation Therapy | No | 164 | 163 | 0.993 | 163 | 164 | 0.165 |
|  | Yes | 4 | 4 |  | 2 | 6 |  |
| Sex | female | 64 | 56 | 0.316 | 58 | 62 | 0.682 |
|  | male | 116 | 127 |  | 124 | 121 |  |
| AFP | ≤400 | 164 | 135 | **<0.001** | 146 | 153 | 0.281 |
|  | >400 | 16 | 48 |  | 36 | 28 |  |
| BMI | <18.5 | 68 | 86 | **0.033** | 23 | 71 | **<0.001** |
|  | 18.5-24 | 35 | 19 |  | 83 | 31 |  |
|  | >24 | 77 | 78 |  | 76 | 79 |  |
| APOA1 | \| ≤17.12 \| \| --- \| | - | - |  | 57 | 125 | **<0.001** |
|  | >17.12 | - | - |  | 125 | 48 |  |
| APOA-1 methylation | ≤0.3147 | 57 | 125 | **<0.001** | - | - | - |
|  | >0.3147 | 125 | 58 |  | - | - | - |
| Recurrence status | No | 94 | 89 | 0.565 | 85 | 103 | 0.067 |
|  | Yes | 88 | 94 |  | 97 | 80 |  |
| Living status | Alive | 109 | 127 | 0.057 | 117 | 119 | 0.882 |
|  | Dead | 73 | 56 |  | 65 | 64 |  |


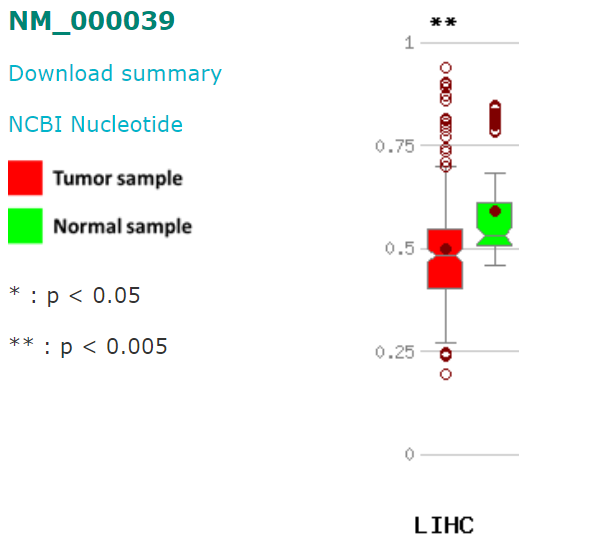


**Figure S****1:** Methylation level of APOA-1 in normal tissues and HCC tissues from public databases. (A) TCGA database; (B) average methylation levels (C) and BOX Plot of number of methylated markers in GSE54503 database.


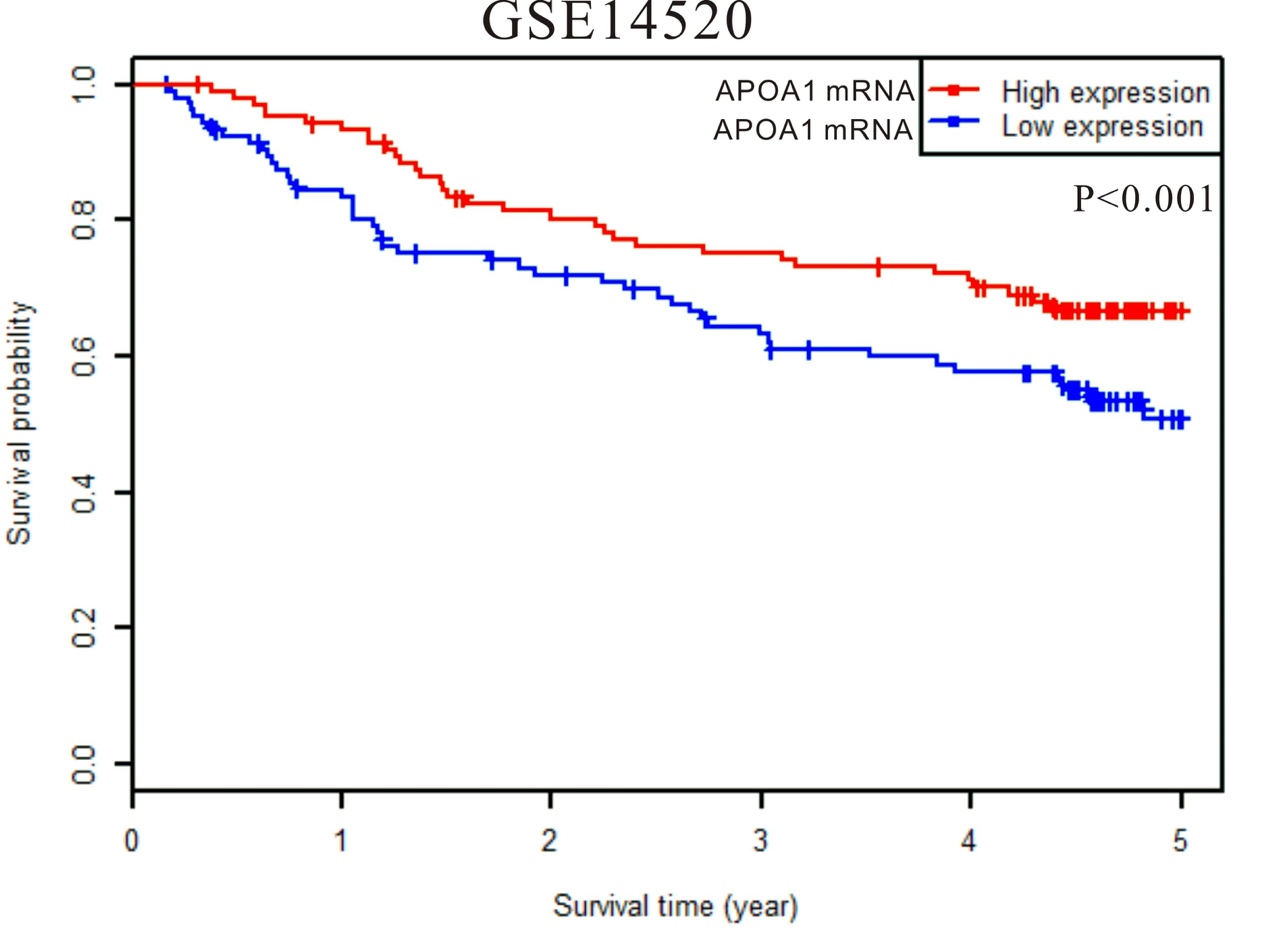


**Figure S2:** Overall survival analysis towards the expression of APOA-1 mRNA was performed in GSE14520(GEO dataset).
